# Editorial Notes

**Published:** 1944

**Authors:** 


### Editorial Notes

**New Professors.**

Dr. J. E. Harris, M.A., Ph.D., Cambridge, B.Sc.London, has been appointed to the Chair vacant by the appointment of Professor C. M. Yonge, to be Regius Professor of Zoology at Glasgow.

Dr. Wilson Baker, Fellow and Tutor of Queen's College, Oxford, has been appointed to the Capper Pass Chair of Chemistry, vacant by the appointment of Professor E. L. Hirst to the Chair of Chemistry at Manchester.

**Grants for Research.**

For the fourth year in succession the Nuffield Provincial Hospitals Trust has made a grant of £1,000 for distribution by the Bristol and District Divisional Hospitals Council.

For the year 1944-5 the Trust has agreed that the £1,000 grant be used as follows:—

(1) £500—for the engagement of a whole-time assistant for Research into the chemoprophylaxis of acute rheumatism.

To this post Dr. D. N. Ross has been appointed. He qualified M.B., Ch.B., at the University of Glasgow, was House Physician in Kilmarnock Infirmary, and R.M.O. in Harrogate Royal Bath Hospital. He joined the R.A.M.C. in 1942, but relinquished his commission on account of ill-health.

(2) £500 to Mr. A. J. Wright for research on Mènière's Disease.

**Work only the Blind can do.**

From the Monthly Bulletin of the Ministry of Health and the Emergency Public Health Laboratory Service for October, 1944. We quote the following:—

Work has been found which only the blind can do—shorthand-typing in the darkness of a radiography room.

A blind typist employed at the Ministry of Health was recently transferred for duties at one of the base hospitals in the Emergency Hospital Scheme under the Department of Health for Scotland. The radiologist there—a leading consultant—found that she was able to solve one of his big problems.

Until now the radiologist has had to memorize his verdict on each examination and make up his records at the end of a series of screenings. The room being in darkness, except for the dim glow of a red lamp at the control table, an ordinary shorthand-typist was unable to take and read notes.

Now the blind typist, with typewriter, sits at a desk in a corner of the X-ray room. When the radiologist has completed each examination, he dictates his findings, which she takes down in shorthand or immediately types out. As soon as the last patient has gone, a neat accurately typed report awaits the radiologist's inspection.

Duty in the dark room over, she assists the radiologist by taking down at his dictation his readings of X-ray films submitted to him by his radiographer. Here again, the notes are often taken down direct on the typewriter. Copies of the reports are then sent to the Medical Officers whose patients have been examined.

This blind typist has summed up her duties in these words :—" I have the unique experience of realizing that, at least in this instance, I am able to perform work which those in possession of their sight would be unable to undertake."

